# Antisemitic comments on Facebook pages of leading British, French, and German media outlets

**DOI:** 10.1057/s41599-022-01337-8

**Published:** 2022-09-29

**Authors:** Matthias J. Becker, Laura Ascone, Hagen Troschke

**Affiliations:** Zentrum für Antisemitismusforschung (ZfA), Technische Universität Berlin, Berlin, Germany

**Keywords:** Language and linguistics, Cultural and media studies

## Abstract

We conducted a cross-national study on antisemitic hate speech on the *Facebook* profiles of leading media outlets in the United Kingdom, France, and Germany. In a combination of qualitative pragmalinguistic analysis and quantitative analysis, we examined their comment sections concerning the conceptual and linguistic repertoire of verbal antisemitism in these three languages as well as to the frequency of antisemitic utterances. The corpus comprises 4500 comments (1500 for each language) made in reaction to the media’s *Facebook* posts reporting on an escalation phase of the Arab–Israeli conflict in May 2021. Since in antisemitism studies, Israel—and issues related to it—are widely perceived as today’s main pretext for communicating antisemitic resentment, unsurprisingly, those events led to the emergence of antisemitic content online. This article contrasts the findings of antisemitism in the three countries’ comment sections and illustrates them by presenting a variety of linguistic realisations of various antisemitic concepts and illustrates the corresponding steps of interpretation.

## Introduction

From 10 May 2021, the Arab–Israeli conflict was marked by an 11-day escalation period. The respective events triggered an enormous increase in media coverage on the conflict, social media campaigns, as well as antisemitic demonstrations, hate speech and violence worldwide. The British charity *Community Security Trust* (CST Community Security Trust, 2021) “reported 639 incidents in May, 49% of the total for the first half of 2021”, a record rise in UK antisemitic incidents. Also in other countries like France and Germany, an increase in such incidents could be observed (SPCJ, 2021; Pau, 2022; RIAS Bayern, 2022; RIAS Berlin, 2022).

The reason for choosing this particular discourse event is the following: With the corpus study, we intend to explore the question of how today’s dominant form of hostility towards Jews—Israel-related antisemitism—is communicated across countries. The escalation phase is a suitable discourse event since the conflict evokes antisemitic attitudes across societies and milieus. Accordingly, all results on the following pages refer to the analysis of threads related to media coverage of the Arab–Israeli conflict.^1^

In order to capture the first online reactions to this period of violence and to the interaction of both parties involved with their respective motives and acts, the measurement period of our analysis is between 10 and 13 May, when preceding tensions tipped over into a belligerent escalation. Our focus was on articles dealing with both Hamas rocket fire and the Israeli army’s retaliation. The study investigates comment threads on the *Facebook* profiles of the leading British, French and German mainstream media outlets. This allowed us to collect a great number of reactions from a wide range of the political spectrum.

This contribution aims at examining the scale of antisemitic content in the comments posted in reaction to the articles dealing with that particular phase of the Arab–Israeli conflict. The specificity of our corpus allowed us to conduct a comparison of the occurrence of antisemitic concepts communicated in the three languages under investigation—and thereby the way applied antisemitic frames differ or are similar. In other terms, by analysing the antisemitic reactions posted in the threads of those *Facebook* profiles, this study aimed at investigating, from a qualitative perspective, how users react to the same trigger in three different countries. It is important, however, to point out that our corpus-based analysis did not aim at realising a social media ethnography (Postill/Pink, 2012) investigating the users’ behaviour and their interactions. Rather, our goal was to illustrate the way antisemitic comments emerge and are expressed. This way, it was possible to see how users may convey antisemitic content in an environment where such hate ideology is generally condemned. Both the content and form of these comments were investigated. As far as the content is concerned, particular attention was paid to the antisemitic stereotypes users refer to. As to their form, a pragmalinguistic approach was adopted in order to examine the way the stereotypes are verbalised (e.g., through allusions, metaphors, speech acts, etc.).

On the following pages, we present our corpus and methodology as well as a short description of those antisemitic concepts that appeared most prominently in the corpus and whose verbal realisations are discussed later on. After this, we give an overview of the distribution of antisemitic concepts—and comments in general—in the three-country subcorpora. Subsequently, in the section “Qualitative findings”, the linguistic characteristics of our corpus will be explored. Here, we will present the results of our qualitative content analysis of the threads examined, that is how antisemitic comments are verbalised by *Facebook* users, not only on the word level (e.g., through the use of puns) but also on the phrase one (e.g., by asking rhetorical questions). The comments we chose for this purpose are examples of the most frequent antisemitic concepts. However, they are not representative of certain types of comments but serve to illustrate linguistic realisations of which there are countless. At the same time, a certain phrase may appear several times in a similar way in our corpora. However, since the corpora are limited in size, we cannot highlight such similarities as recurring patterns, as it could be a coincidental accumulation.

## Research design

### Corpus

The goal of this analysis was to investigate antisemitic hate speech in comment sections of mainstream media. This choice shifts the focus away from radical fringes of the political spectrum and is in line with the fact that antisemitism is communicated throughout society—and stresses the necessity to understand patterns of antisemitism in those discourse spaces. Therefore, the leading British, French and German media outlets were selected to build our corpus. As far as the British corpus is concerned, the analysis was conducted on *BBC*, *Daily Mail*, *The Guardian*, *The Independent*, *The Spectator*, *The Telegraph*, *The Times*, *Daily Express*, *Daily Mirror*, *Financial Times*, *Metro* and *The Sun*, while the French corpus was collected from *Le Monde*, *Libération*, *Le Figaro*, *Le Parisien*, *Le Point*, *L’Express*, and *20* *min* As to the German corpus, the study was conducted on *Bild*, *FAZ*, *Focus*, *n-tv*, *rp-online*, *Der Spiegel*, *Süddeutsche Zeitung*, *taz*, *Die Welt*, and *Die Zeit*. The study focuses on threads on the *Facebook* profiles of these media. This allowed us to overcome the limits set by paywalls and the deactivated comment sections of certain media outlets and to collect a great number, and wide range, of users’ reactions. In order to make the datasets comparable and subject them to consistent qualitative corpus analysis, we applied a rule-based selection of *Facebook* threads and comments. For threads to be relevant for the corpus the corresponding *Facebook* post had to relate to Hamas rocket fire or the Israeli army’s retaliations. In the case of the German subcorpus they combined both. Depending on the number of threads available in each of the languages 100–150 comments were coded (following the chronological order in the thread) in order to achieve an evenly distributed number of comments throughout the threads of a subcorpus.

### Methodology

Due to its high level of acceptance, we use the working definition of the International Holocaust Remembrance Alliance (IHRA) as a basis for the identification of antisemitic posts.^2^

Yet because of the IHRA’s constricted (even though flexible) form, it still requires additional complexity in order that it can be utilised as an analytical tool in web communications research. The extensive operationalisation resulted in a detailed list of 57 stereotypes and topoi—considering the main components of anti-Judaism of the Middle Ages, racial antisemitism of early modern times, secondary antisemitism after 1945 as well as Israel-related antisemitism—according to which antisemitic utterances can be precisely categorised on the content level. Therefore, the list consists of classical stereotypes (such as evil^3^, power, greed, child murder^4^ and more contemporary attributions (such as instrumentalisation of the holocaust, nazi analogy, denial of israel’s right to exist) (cf. Schoeps/Schlör, 1995; Julius, 2010; Nirenberg, 2013; Schwarz-Friesel/Reinharz, 2017; Becker, 2021).

The IHRA definition is not without its critics—indeed the question of its adoption by states, local government and other organisations has been subject to intense political debate and struggle in a number of countries over the past decade or more. Authors of the Jerusalem Declaration on Antisemitism (JDA)^5^, for example, argued that the guidelines for IHRA needed to be clearer and that it would focus too much on Israel while being unable to clearly draw the line between criticism and antisemitism. However, this is something the JDA does not achieve on its part. To name just two instances that it does not capture: the accusation of apartheid gravely misinterprets the reality in Israel and serves to demonise it. A comparison between Israel and National Socialism is essentially antisemitic because of its demonisation and the inherent victim-perpetrator-reversal (cf. Becker, 2021).

In addition to the content-related phenomena covered by recourse to research on antisemitism, we drew on linguistic and discourse-analytical research in order to better understand the language and imagery through which antisemitic ideas are conveyed online today.^6^ Antisemitic stereotypes represent mental units that ever since have been handed down through language and images, sometimes over centuries.

Since antisemitism in the politically moderate web milieus we studied collides with culturally developed, collective self-images—resulting that web users who want to communicate antisemitic ideas run the risk of being sanctioned—it is all the more important to take implicit patterns of language use into account. That is, users rather do not communicate an antisemitic stereotype explicitly, but they choose communicative detours through which the same concept of devaluation and exclusion remains, but its presentation appears concealed (cf. Schwarz-Friesel/Reinharz, 2017) with a high variety of forms of verbalisation. This is the reason for our decision to conduct qualitative content analyses in order to assess forms and frequencies of antisemitism online.^7^ Due to the perennial observation that, in segments of the political mainstream, antisemitic derogation and/or exclusion generally take place implicitly, searches for problematic words (such as slurs, threats, death wishes, explicit reproductions of stereotypes that appear in far-right contexts, for example) would not be able to lead us to those posts in which antisemitism is communicated. Thus, quantitative analyses that follow a rather limited understanding of manifestations of hate speech cannot be the means of choice. Even vector analyses (in which accumulations and combinations of words are investigated within a medium and reference period, i.e., researchers are guided by corpus-specific frequency distributions and not by predetermined, deductive categories (e.g., ADL (Anti-Defamation League), 2019; Zannettou et al., 2020) cannot capture the numerous comments in which antisemitic stereotypes are reproduced without recourse to relevant-specific word selections.

In other words, qualitative analyses impressively demonstrate that comments sections where a predefined search for deductive categories resulted in few or no hits can nevertheless contain large numbers of antisemitic statements; they also illustrate that the constitution of antisemitic attributions can be semantically so open that conspicuous word accumulations—or even relevant terms—can be completely absent.

In order to organise the numerous patterns of implicitness and bring them together with the observations of antisemitism studies, we draw on the research of pragmalinguistics—a discipline that focuses on the context-sensitive understanding of language use (cf. Levinson, 1983, 2000; Reisigl/Wodak, 2001; Meibauer, 2013, 2019; Schulze/Pishwa, 2015; Schwarz-Friesel/Reinharz, 2017).

In the corpus study presented here, we examined antisemitism in reader comments following Mayring’s qualitative content analysis (2015). While conducting the qualitative examination of web comments, we developed the categories deductively as well as inductively both in relation to antisemitic concepts (including stereotypes in particular) as well as to the linguistic and visual phenomena employed by web commenters. Especially in the extrapolation of country- and milieu-specific debates, we follow an inductive category formation in order to include any novel attributions (as well as their distribution in the individual web milieus).

The categorisation and qualitative analysis of the corpora is being conducted with the analysis tool MAXQDA. The code system drawn up in this tool covers the diversity of conceptual, semantic/pragmatic as well as image-related phenomena that accompany the object of investigation.

## The conceptual basis and frequency of antisemitism online

### Frequently appearing antisemitic concepts

The stereotype evil is one of the most general antisemitic concepts. It implies that Jews or Israel and its politics or actions are characterised by essential wickedness, or the desire to cause comprehensive damage to others in a targeted and proactive manner. It can boldly occur by the attribution of such a trait or in various ideas that incorporate adaptations of the stereotype, e. g. Israel being a terrorist state.

Child murder—or blood libel—is an antijudaist trope according to which Jews allegedly killed Christian children in order to use their blood in religious rituals. A popular modern adaptation of this stereotype is the claim that Israel is deliberately killing children in the Arab–Israeli conflict.

The nazi analogy makes an equation between Jews or Israelis and Nazis. It can be realised via comparisons of traits, actions, persons, places, etc. or by allusions hinting to the realm of National Socialism.

The stereotype of (disproportionate) jewish/israeli influence on the media represents the idea that Jews or Israel would have partly or absolute control of the media.

The topos of a taboo of criticism asserts that freedom of expression would be curtailed when it comes to issues concerning Jews and/or Israel.

The idea of a sole guilt of israel for the arab-israeli conflict assigns the general responsibility for the conflict—and for being attacked—to Israel. (It is an adaptation of blaming jews for antisemitism.) This blanket attribution combines the stereotype of Jews as troublemakers with ideas of an inherent aggressiveness.

denying jews the right to self-determination in their own state can for instance be expressed by targeting or denying Israel’s legitimacy or negating it altogether.

The apartheid analogy in reference to Israel alleges that a system of structural racist oppression would exist in Israel. A similar and more general concept is that of Israel being a racist state—without connecting it to a specific historic scenario.

Closely related to those concepts is the colonialism analogy. By equating Israel to past colonising states or their actions it asserts that the state of Israel would be a colonialist enterprise itself or that it would occupy the West Bank for colonialist reasons – the former deligitimising the sovereignty of Israel.

### Quantitative distribution of antisemitic user reactions

The recent escalation in the Arab–Israeli conflict triggered extensive coverage across all three countries and generated a consistent number of antisemitic responses in social media. However, the results of our analyses demonstrate that these vary greatly between countries. The analysis of *Facebook* pages of leading media outlets in the UK reveals a frequency of antisemitic statements twice as high as in the other two countries: 26.9% of 1504 analysed comments. 12.6% of 1500 comments on *Facebook* pages of French mainstream media were antisemitic—almost the same share as for leading German media outlets: 13.6% of 1520 comments.

There are various reasons for the high presence of antisemitism in the comment sections of British media. On the one hand, due to the prevalence of English around the world, posts in British media attract an international audience, which possibly contributes to the spread of antisemitism.^8^ On the other, within British society, awareness of antisemitism has generally been relatively low. One has not been dealing with Jew-hatred in the past; its domestic dangers were often disregarded (cf. Julius, 2010). Instead, the focus was on the colonial heritage and the various forms of racism. This lack of awareness is perhaps one reason for what Robert Wistrich (2011, p. 16) has described as Britain’s “pioneer[ing]” role in the construction and proliferation of Israel-related antisemitism since the 1960 s. The security supposedly provided by the UK’s modern history with regard to Jews has contributed to an atmosphere characterised by much greater acceptance of—or failure to recognise or interrogate—antisemitic speech and behaviour with regard to Israel than would be tolerated, at least on the surface, in Germany.

For 38.7% of the antisemitic comments in the UK corpus, the thread’s context was decisive for inferring the hidden meaning. The most frequently communicated concepts were, in this order, evil (39.8%), israel’s sole guilt in the conflict (27.9%), child murder (8.1%), the denial of jewish self-determination (7.7%), apartheid (5.2%) and nazi (4.2%) analogies, as well as amorality (4%).

The analysis conducted on the French corpus reveals that around 62% of the antisemitic comments required the wider context of the thread to be taken into account in order to determine their antisemitic character. Almost half of the antisemitic comments present the evil stereotype (46.8%). Other antisemitic concepts French users evoke most often are denial of jewish self-determination (17.8%), colonialism (13.1%) and nazi analogies (7.8%), child murder (11%) and amorality (6.3%).

For the German comments sections, 48.3% of the antisemitic meanings could only be inferred via the context. The most frequent antisemitic attributions were those of evil (41.0%), israel’s sole guilt in the conflict (10.1%), jewish/israeli influence on the media (8.2%), a taboo of criticism towards Israel (8.2%), the apartheid analogy (6.2%), child murder (5.8%), and denial of jewish self-determination (5.3%).

It is striking that attributions towards Israel of being essentially evil or committing major evil have been by far the most frequent in all three countries—that is to say that Israel is principally connected to a range of demonising evaluations that are regularly repeated and shared across the countries. The evil stereotype serves as the basis for further topoi, be it by means of the depiction of Israel as a nazi or apartheid state, or the sole culprit in the conflict. The conceptualisation of Israel as the last existing colonial state plays a greater role in the French corpus than in its British equivalent. Inversely, the accusation of an apartheid regime as well as the concept of israel’s sole guilt in the conflict is less present in the French debates.

Moreover, there are two shared dominant topoi in all three country discourses: child murder and the denial of jewish self-determination. The former evidently continues to serve as a perennial mode of antisemitism. The latter ties in with the antisemitic conceptualisation of Israel as such: the end of Israel’s existence one way or the other, with the foreseeable catastrophic consequences for its Jewish population.

The topos of taboo of criticism is far more prominent in Germany than France or the UK. This difference may be due to the centrality within German antisemitic discourses of the idea that German consciousness of guilt for the Holocaust has made Jews virtually untouchable, whether through social desirability or the influence of an (un)determined power, and that both this guilt and untouchability should be rejected. There is also relatively little appearance of the stereotype of jewish/israeli influence on the media in the UK and France. With regard to the UK, the majority of commenters who dismiss the image of Israel presented in the British media do so solely by accusing the latter of a pro-Israeli bias (40.7% of all comments). This accusation is compatible with the notion of a jewish influence on the media—an interesting contrast to this more overt allegation found in the British corpus.

These numbers shed light on both the differences and similarities in the way *Facebook* users reacted to the escalation phase in the Arab–Israeli conflict in the three countries under investigation. However, a larger amount of data would have been necessary in order to conduct quantitative analyses and then provide statistically significant results. It is for this reason that for this study only a qualitative approach was adopted and whose findings will be presented in the following section.

## Qualitative findings

### British corpus

The analysis of the British corpus focuses on the first 150 comments of ten threads that were taken from the *Facebook* profiles of *The BBC*, *Daily Express*, *Daily Mail*, *The Financial Times*, *The Guardian*, *The Independent*, *The Mirror*, *The Spectator*, *The Telegraph*, and *The Times* (cf. sources).

The UK media’s comment sections bring to light that commenters frequently direct various antisemitic stereotypes against Israelis (and Jews)—whether linguistically or via imagery (or both). Among the predominant ones, the evil stereotype stands out, especially when the articles focus directly on Israel’s reaction to the Hamas rocket fire.

In the first example presented here, the notion that Israel represents the evil of the world is expressed by a dichotomy between Israel and the world: “Th[is] country throw[s the] world into war” (BBC-FB[20210511]); “Israel is a disgrace and criminal! America should stop this if they want to win respect from the world!” (Tim-FB[20210511]). In other comments, the very end of Israel is equated with global peace: “end Israel and all the world..not just Palestine will find peace” (DM-FB[20210512]; see also comparable references to Zionism: “Remove zionism from the pages of history and peace will return,” Tim-FB[20210511]).

Regarding negative ascriptions of this kind, it may be argued that only Israel or the Israeli state is meant. The following example, however, illustrates that a line is not drawn between the state and its population: “Same old. Israelis using 21st-century technology supplied by the West to slaughter Palestinians and steal their homes” (BBC-FB[20210511])—nor do commenters distinguish between Israelis and Jews that both are characterised as malicious and wicked: “the one and only thing which unites Israeli Jews is their destruction of others, especially of Palestinians” (Spe-FB[20210512]).

The evil stereotype can also be expressed implicitly, e.g., via wordplay (“israhell,” Tel-FB[20210511] or—referring to the then Israeli prime minister—“Satanyahu,” Tim-FB[20210511]), demonstrate the conceptual proximity to the devil stereotype. Also dehumanising metaphors that reproduce age-old visual representations of Jews can be traced in the British corpus (e.g., “ziopigs”, Tel-FB[20210511]).

As mentioned in chapter 3.1, the evil stereotype—like all other topoi of antisemitism – has adapted to new communicative conditions over time. Accordingly, web users may present Israel as a strikingly evil force by enriching it conceptually and framing the country as a criminal or even terrorist state: “built by brutal gangsters” (Gua-FB); “Zion is built on terrorism” (FT-FB[20210511]); “terrorists = IsRealHell” (Spe-FB[20210512]).

Another popular form of demonising, devaluing and marginalising Israel in today’s discourse is its equation with Nazi Germany. The nazi analogy occurs in various forms in the corpus. Explicit forms are direct equations (“Zionism = Nazism,” Gua-FB) or overt references to the historical perpetrators (“Nazis under a different flag,” Mir-FB[20210512]). In both cases, the equal sign, as well as the phrase under a different flag, serve as a connective substitute (similar to *remind me of*) through which the classic construction of comparison, based on the scheme X is *like* Y, gets modified (cf. Becker, 2021, 221 ff.).

Often, the nazi analogy is implicitly justified by accusing Israelis of ignorance regarding history: “The Israelis have so quickly forgotten how they were treated by the Germans!,” Tel-FB[20210511]). Based on habitualised phrases, such comments imply that Israelis are now carrying out the same atrocities that once happened to them—a subtle variant of the victim-perpetrator reversal which the nazi analogy is part of.

Writers can also activate the analogy more subtly, through the use of allusions. Doing so, they conceptualise the current Arab–Israeli conflict without having to specifically refer to the historical era or its perpetrators and victims (cf. Becker, 2021, 249 ff.): “[the Israeli] government roll[s] out an extermination plan” (Tel-FB[20210511]). *Extermination plan* is a term strongly linked to the plans of the Nazis to annihilate the Jewish population of Europe. The same holds true with regard to the word *resistance* in particular contexts in order to refer to Hamas, like in the following rhetorical question: “Terror groups? [Was] the French resistance a ‘terror group’? =” (Spe-FB[20210512]). In other cases, names standing for Nazi Germany are modified so that readers can infer the allegation that Israel represents the continuation of Nazi crimes: “Fourth Reich Rising” (Tim-FB[20210511]).

Other commenters use references to the Nazi scenario, not to equate Israel with it, but again to allude to the evil character of Jews and by doing so, to justify Nazi crimes: “the big dùde of Germany (Hïtłèr) once saīd that “ he côuld’ve kįlléd em all, but hė left some so people can know why he did that” (Tel-FB[20210511]).

Next to references to Nazi Germany, commenters regularly equate Israel to apartheid South Africa or depict it more generally as a racist state and/or society: “Above all, Israel is an apartheid based country that has been occupying Palestine since 1948” (Ind-FB[20210511]). Next to the distorting usage of the term *apartheid*, the comment exemplifies the tendency among users to not just rejecting the settlements in the Westbank only, but the existence of the (1948 founded) Israel per se.

It is interesting to observe that even in comments that criticise the Palestinian side, the apartheid analogy (alongside colonialism analogies) is used to argue for the renunciation of terror on the Palestinian side: “If I were being sanctimonious, then I’d say that the Palestinians have had multiple examples of successful non-violent self-determination movements to draw on—Gandhi, MLK, Mandela—but they’ve instead opted for a self-destructive policy of zero-sum eternal war with Israel. How’s that working out for them in a world where India and South Africa are independent sovereign nations?” (Spe-FB[20210512]). Even though it is a comment that suspends the constant rejection of Israel in the comment sections studied, demonising attributions are nevertheless realised here through onomastic allusions (*Mandela* alluding to South Africa, *Gandhi* to colonised India).

The frequently used, alternating references to colonialism or apartheid are complemented by ascription concerning the opponent of the conflict. By means of the acronym “PLM” (Gua-FB and Ind-FB[20210511]), users allude to the *Black Lives Matter* movement, i.e., by means of a changed label, the Hamas–Israel conflict is subtly placed in the context of racism in Western societies. In this way Israel is accused of structural discrimination against Palestinians, ignoring the complex genesis of the conflict, as well as the role of Islamism: “This is the equivalent of […] blaming George Floyd that he stopped breathing under the knee of (yet another) militant police officer” (FT-FB[20210511]); cf. also multiple statements such as “At least 9 children were killed in Gaza.. but yeah they are brown” (FT-FB[20210511]), or “Israelis hate black people,” (Ind-FB[20210511]).

In line with many such allegations, the ongoing confrontation between the conflicting parties is perceived as the sole cause of the Israeli side: “The entire blame is on Israel. Once they top their continued 7-decade aggression there will be no need for a resistance,” (DM-FB[20210512]); “If I didn’t want rockets fired at me, I would simply not set up a murderous apartheid settler colonial state that oppresses Palestinians on a daily basis,” (Ind-FB[20210511]); “Simply put: Zionism is the problem” (BBC-FB[20210511]). Given that the Arab–Israeli conflict today is the main trigger for antisemitic resentment worldwide, the relation to the stereotype’s classic equivalent (that jews are to blame for antisemitism) becomes apparent. The following comment subtly expresses the idea that Jews themselves are to blame for the hatred they have been subjected to ever since: “I wonder why the British government sent the jews away to philistine and didn’t keep them in its own land >” (BBC-FB[20210511]).

Other examples present comparisons that foreground a disproportionate, highly unjust relationship—closely linked to scenarios of violence (“as perverse as Mike Tyson punching a toddler,” FT-FB[20210511]), murder (“Monster VS children,” DM-FB[20210512]) and even rape: “you mean those homemade rockets […]?You know how vicious fingernails can be? Have you SEEN the harm done to rapists by their victims nails raked over their faces, even when the rapists smash in the victims head with a hammer? Ohh.. those vicious and nasty nails” (Spe-FB[20210512]). By means of these comparisons, commenters create an even more emotionally charged interpretative framework that clearly opposes empathetic emotions for the Israeli side, in which they unambiguously identify the guilty party in the conflict and relativise (or even negate) the major escalating aggression that is reflected in the large number of rockets fired at Israeli civilians.

The allegation of Israel’s evil nature and sole guilt in the conflict is also presented in combination with slurs and the denial of the jewish state’s right to exist: “It’s not Israel Palestine until 1948 then these scums came begging for safety and bit the hand that fed them” (Mir-FB[20210512]).

The antisemitic idea of Jews being immoral, hypocritical and deceitful, which has already been echoed in the latter example, is further developed in many other comments. Here, users conceptualise Israel as a state that sees deceit and obfuscation as acceptable means for its own advancement. E.g., users accuse Israelis of presenting themselves as victims in order to generate political capital out of their deliberate continuation of the conflict, and suggest they are willing to accept civilian casualties on the Palestinian side in order to do so: “Israels government is doing this on purpose to try and boost the ailing election ratings of the evil man in power at the moment” (DE-FB[20210512]) and “No one fired rockets stop the lies! They do it every year same time, see the pattern” (Gua-FB). Commenters regularly claim that they speak for the world when they accuse Israelis of their evil character, their lies and instrumentalisation of antisemitism: “The world is sick of their hate and their ‘victim’ mentality” (Gua-FB).

The allegation of a lie encompassing the whole world is also intertwined with the allegation of favouritism on the British media’s side towards Israel and Jews—an accusation regularly made against both left-liberal and conservative media outlets. In this context, users imagine a jewish-zionist influence on British media (“Hypocrisy at its finest from the Daily Mail. Owners are in the pockets of the Zionists, no surprise,” DM-FB[20210512]; “Utterly Disgraceful reporting at best. A dog doesn't bite his Master I guess” FT-FB[20210511]; “Who owns the British media??,” Mir-FB[20210512]; “‘Independent’ my a**. You are bought by the zionist lobby. Sheep’s!,” Ind-FB[20210511])—sometimes to the extent that media are directly conceptualised as Zionist (“Guardian of Zion,” Gua-FB), with antisemitic stereotypes such as hypocrisy and deceit (or even greed) then transferred to them—and subsequently to Israel: “Nice try zionistNow make money n more bias news” (FT-FB[20210511]).

These distorted images are in part integrated into notions of a Jewish conspiracy that relate directly to conditions on the ground. For example, users suggest that Israel itself supplies Hamas with rockets in order to deliberately stoke the conflict: “how are hamas able to get rockets or any military weapons when Israel controls everything that goes in and out of Gaza?” (Tel-FB[20210511]).

Also, users complemented such imputations by the accusation of a secret arrangement between Israel and the British royal family, put forward to manipulate public opinion about the conflict (“I guess the British Royal family-owned news outlet doesn't want us to know the real story,” DM-FB[20210512])—or they ultimately refer to a more general global Jewish conspiracy (“Jews rule the WORLD!,” FT-FB[20210511]).

### French corpus

The French corpus was composed of the first 100 comments posted in reaction to 15 different articles from 7 mainstream media outlets: *Le Monde*, *Le Figaro*, *Libération*, *Le Parisien*, *Le Point*, *20* *min*, and *L’Express*.

Focusing on Hamas rocket fire and IDF’s retaliation, these articles elicited hostility towards Israel and Israelis rather than Jews. Our analysis clearly shows that, nowadays, classical antisemitic stereotypes are adapted to the current situation. For instance, as we will see, the devil stereotype tends to be associated with the Israeli government’s actions against Palestinians rather than with the old belief that Jews would adhere to the Anti-Christ.

Although the French corpus presents fewer antisemitic comments than the English one, the way the targets of these antisemitic comments are presented is quite similar in the two corpora. Like in the English threads, the idea that Israel and Israelis are evil entities constitutes the most frequent stereotype. This representation is exaggerated and nuanced in different ways, according to the specific event it refers to. The more condemnable the action is to the eyes of the user, the more emphasised the evil attributions are. This is shown in a comment posted in reaction to an article from *Le Monde*: “it’s the only people in the world boasting of killing human beings” («C’est le seul peuple au monde qui se vante de tuer des êtres humains.» Mon-FB[20210511]). The user, who is likely to refer to the title of the article “Israel claims to have killed 15 members of Hamas and of the Islamism Jihadi in Gaza”, condemns not only the killing of human beings but also the fact that Israel would boast of this. This double condemnation is exaggerated by the speaker through the use of the hyperbola “it’s the only people in the world”, which would mean that Israel is considered the evil people par excellence. The exaggeration is operated by the user also through the generalisation of the whole Israeli/Jewish people. Furthermore, this characterisation of Israel as evil is not presented by the user as their point of view. Rather, it is exposed as a general and, therefore, incontestable, truth.

A different sort of exaggerated generalisation is the attribution of terrorist state to Israel. Out of the 1500 comments analysed, 32 present and define Israel as a terrorist state, like in the following example: “A terrorist, racist, homophobic state that kills children, women and handicapped persons” («Un état terroriste, raciste, homophobe, tueurs d’enfants, de femmes et de personnes en situation d’handicap», Lib-FB[20210512]). In this example, the user lists the alleged condemnable actions of Israel in order to emphasise its evil character. Resorting to the use of lists constitutes a discursive strategy allowing the user to exaggerate their claim. In this example, the user then considers Israel as a terrorist state. As a consequence, the position of Israel is diminished because of its alleged terrorist actions. In other terms, to the eyes of the user, Israel becomes closer to its antagonist in the conflict, that is the terrorist organisation Hamas, than to any other democratic state. However, the fact that in these comments users still employ the word “state” shows that Israel is considered as such by the user and, therefore, its existence as a state is not denied. Yet, the attribution of terrorist state to Israel, like to any other country, could constitute a form of delegitimisation of its position.

On the contrary, the interpretation of the following comment could be slightly different: “These are victims of the Israeli terrorism, civilians, children, women, elders… Israeli terrorism has no limit” (« Se sont des victimes de terrorisme israelien, des civiles, des enfants, des femmes, des agés…le terrorisme israelien n’a pas de limites », Mon-FB[20210512]). Contrary to the last example, in this comment, the user refers to “Israeli terrorism”, which prevents us from knowing whether Israel is recognised as a state by the user or if its existence is denied. However, a similarity between these two comments can be identified in the way resort to lists in order to emphasise their statements. Furthermore, in this last example, the user employs the ellipsis (“…”), which underlines the fact that the list is even longer. The exaggeration of the characterisation of Israel reaches the pick in the last sentence “Israeli terrorism has no limit”).

In 4 comments, the users resort to the means made available by computer-mediated communication: “Isra-Hell = terrorists” («Isra-Hell = terroristes», Fig-FB[20210510]). In this comment, the user presents Israel as a terrorist state not through verbal expression but rather through visual elements made available by computer-mediated communication, in this case the symbol “=”.

A discursive feature characterising these comments is the fact that through the argumentative maxim of the act (Plantin, 1993), according to which the quality of a person depends on their behaviour, the speaker transfers the judgement of the action to the actor themselves. More precisely, in this context of the escalation phase of the Arab–Israeli conflict, because of their actions against Palestinians, the Israeli population is devalued, as shown in the following example: “The figures speak for themselves, and Israel is a terrorist people killing women and children with impunity” («Les chiffres parle et Israel est un people terroriste tuant des femmes, des enfants, sans impunité», Par-FB[20210511]). In this example, the killing of women and children makes, in the eyes of the user, Israelis terrorist people. Furthermore, through the argument from authority (Ducrot, 1984), the user refers to figures in order to give weight to their statement.

As stated before, considering and presenting Israel as a terrorist state is a form of generalisation since the user transposes the actions undertaken by a certain government to the entire population.

A more indirect way to present Israel or Israelis as evil entities is through the analogy between Israel and the Nazi regime. Most of the time this nazi analogy is activated by comparing, more or less indirectly, the violence faced by the Palestinians in the current conflict and the Jews in the Nazi scenario. However, this analogy may be presented in different ways. In a thread from *Le Parisien*, a user describes Gaza as “a new open-air concentration camp” («un nouveau camp de concentration à ciel ouvert», Par-FB[20210511]). Here, the expression “concentration camp” clearly alludes to the conditions the Jews had to face during World War II under the Nazi regime.

On the contrary, in a thread taken from the Facebook page of Le Monde, another user refers to a previous comment stating: “The same discourse as Nazis’ when Jews killed German soldiers” (Le meme discours qu’avaient les Nazis quand les juifs tuait des soldats allemands”, Mon-FB[20210511]). The previous comment this user refers to claimed that in the Arab–Israel conflict, the latter is just defending itself against the attacks of the Palestinians. Even though this comment might appear as not antisemitic, a more accurate reading of this nazi analogy reveals the antisemitic character of this statement. More precisely, according to this user, Nazi and Israelis are implicitly presented as perpetrators who would justify and, as a consequence, legitimise, the murder of the respective victim group (i.e., Jews in the Nazi scenario and Palestinians in the current one) by using the same argumentative strategy.

Furthermore, in some of the comments that we analysed, the analogy was operated through the process of the denomination. In these cases, by using compound words, the user designates their target (in this case, Jews or Israelis) as Nazis like in the comment “the Nazi-Zionists” («les Nazi-Sionistes», Lib-FB[20210512]). This process of denomination intensifies the nazi analogy since, according to the user, two originally separate and distinct groups, that is Nazis and Zionists, form a single and merged entity.

Contrary to the English corpus, the French one is characterised by a different analogy too: the colonialism analogy. Here, Israelis are perceived and presented as colonisers and, more precisely, as foreign people who occupy the territory of Israel, as shown in the following comment: “A land that is being stolen by new settlers coming from Europe” («Une terre qui se fait volé par de nouveaux colons venant d’Europe», Mon-FB[20210511]). The fact that they are occupying this territory implies that their presence there is illegitimate. As a consequence, this analogy is often linked, more or less directly, to the stereotype according to which Israelis are foreigners as well as to the denial of israel’s right to exist. As far as the latter is concerned, Israel may be presented as an illegitimate state; according to a comment published on the Facebook page of *Le Parisien*, “Israel is an illegitimate and illegal state” (Israel est un etat illégitime et illegal, Par-FB[20210511]). However, sometimes the existence of Israel is simply not recognised, as the following comment shows: “your rogue state doesn’t exist” («ca n’existe pas ton état voyou», Mon-FB[20210512]).

### German corpus

The two conflict events at the beginning of the Hamas–Israel conflict—the rocket fire on Israel from Gaza on 10 May and the subsequent bombing of Hamas targets by the IDF—were jointly discussed in one article by each of the observed leading German media outlets, each in a linked article posted on *Facebook*. Web users thus had information on both events and the connection between them and they were able to incorporate them equally into their assessment and evaluation of this conflict phase. The dual focus of the reporting meant that online commentators were not influenced by one-sided coverage but responded to stories describing actions taken by both sides of the conflict. With this approach, we were able to investigate reactions that, in addition to possibly already established attitudes, are based on the reception of both events. A total of posts, 1520 user comments were analysed. Within these comments, the question of the blame for this escalation and attacks against the media for their alleged bias in favour of Israel cropped up with particular frequency. The antisemitic posts were mainly aimed at Israel and Israelis, though in some cases also at Jews. Selected antisemitic ideas are presented here with examples. One concept area revolves around the stereotype evil and includes the stereotype child murder as well as the nazi analogy.

When the evil stereotype is expressed, Israel is often explicitly branded as a *rogue* or *terrorist state*: “Nothing more than a rogue state” [“Nichts weiter als ein Schurkenstaat”] (NTV-FB). Alongside the imputation of malicious activity, this attribution also delegitimises Israel’s statehood. As “the spawn of Europe” [“die Ausgeburt Europas”; a variation of a German idiom in which hell or devil stands in place of Europe] (NTV-FB), Israel is portrayed as the embodiment of all that is negative in the world. However, key terms and explicit attributions are not usually present. The comment “I think when all Israelis come back to Europe and America then we’ll have peace in all Arab countries, no refugees will come to Europe and America” [“Ich denk wann alle Israelische kommen wieder nach Europa und Amerika, dann wir haben Frieden im alle Arabisch Länder kommt keine Flüchtlinge nach Europa und Amerika”] (NTV-FB) makes the absence of Jews in Israel a condition for peace in the region. In so doing the comment also asserts, conversely, that Israel is allegedly responsible for all regional conflicts and thus all flights of refugees from the wider Middle East. In another aspect of the evil stereotype Israelis are accused several times of having an inherent tendency to violence—one at times said to have been temporarily hindered by the Covid-19 pandemic: “They have fought off covid and now they are gaily shooting all over the place again. >” [“Corona ist bei denen bekämpft und jetzt wird wieder lustig rumgeschossen. >”] (B-FB[20210510]).

The stereotype child murder appears—apart from that term—in other explicit ways: “Israelis deliberately kill children and dance while they’re doing it” [“Israelis töten gezielt Kinder und tanzen dabei”] (SP-FB[20210511]); “This is exactly what the Israelis have been waiting for, Jewish bombs are back to executing countless civilians and children” [“Genau darauf haben die Israelis gewartet, jetzt werden wieder etliche Zivilisten und Kinder mit jüdischen Bomben hingerichtet”] (SZ-FB[20210510a]). In both comments, *dance* and *waiting* suggest a wickedness in Israelis that craves bloodshed. Designating the bombs as *Jewish* extends the attribution to the original targets of the stereotype. Other comments, however, require a certain amount of deduction—like the following based on the same premise as the one alleging violent traits: “Covid in Israel over, now back to everyday life, in thoughts with the killed children” [“Corona in Israel vorbei jetzt wieder Alltag in Gedanken bei den getöteten Kindern” (NTV-FB). It invokes the stereotype by presenting the killing of children as an everyday feature of Israeli life. The rhetorical question “So shooting children in the head is legitimate?” [“Kopfschüsse bei Kindern ist also legitim?”] (SZ-FB[20210510b]) contains the presupposition that such crimes would indeed take place. By simply negating the legitimacy one would still accept the presupposition. This is what makes that rhetorical strategy so effective.

The nazi analogy is often verbalised below the line of direct equation of the actors. For expressing it the following comment uses comparison and allusions. The reference to *crimes against humanity*, a category of international law created in response to the Nazi atrocities, is an allusion to Nazism which brings Israel into conceptual proximity with it. It serves as a bracket both for a comparison on the level of action, with Israel said to be re-enacting these atrocities, and for the allusions *deportation* and *ghetto* which once again reinforce the analogy.

“Israel’s policy has been a crime against humanity since the founding of Israel, although they should know what the Germans did to them, that is exactly what they are enacting. The Palestinians are forcibly deported into ghettos with no way out”

[“Israels Politik ist seit der Gründung Israel, ein Verbrechen an die Menschheit, obwohl sie es wissen müssten, das was die deutschen ihnen angetan haben, genau das leben sie da aus. Die Palästinaner werden zwangs depotiert in irgendwelchen Ghettos ohne Ausgang”] (FAZ-FB[20210511]).

An even more indirect allusion evokes that analogy by reference to Sophie Scholl, a resistance activist against National Socialism: “Since its foundation, Israel has only been discriminating and oppressing. You have to fight back. Just like Sophie Scholl fought back then” [“Israel ist seit ihrer Gründung nur am benachteiligen und unterdrücken. Da muss man sich wehren. Genau wie sich Sophie Scholl damals gewehrt hat“] (SP-FB[20210511]). This allusion—emphasized with the comparison *like*—implies that Israel would have similar characteristics as National Socialism and should therefore be countered.

Alongside evil, the second main conceptual area in the corpus relates to the idea of supposed Israeli influence on media reporting and public opinion. It includes the stereotype of jewish/israeli influence on the media and the topos of a taboo of criticism against (in this case) Israel. Conceptually similar to both is the frequent accusation of media bias (motivated either by the media themselves or some unknown cause) which we have not, however, categorised here as antisemitic. A variant of uttering the former stereotype explicitly is this comment: “Since the entire media landscape in Germany is dependent on the money supply by the Zionists, an objective reporting hardly exists.” [“Dia die gesamte Medienlandschaft in Deutschland, am Geldhahn der Zionisten hängt, ist eine objektive Berichterstattung kaum vorhanden.”] (Z-FB[20210512]). But in the analysed discourses the formulations are usually implicit as in the next examples. Based on a rhetorical question that assumes the presence of Israeli propaganda from the outset, the suggestion of a name change implies that the publication is in the service of Israel. A metaphor is then used to depict its supposed relationship to Israel as one of subservience and dependency:

“Why are you doing Israeli terror propaganda? […] Maybe you should be renamed Israel Post Rheinische Post […] YOU are like their dogs that have to obey.”

[“Warum macht ihr Israelische Terrorpropaganda? […] Vielleicht sollte man euch doch umbenennen in Israel Post Rheinische Post […] IHR seid wie ihre Hunde, die gehorchen müssen.”]

To A’s critical question, “Who controls the German media habibi” [“Wer kontrolliert den die deutschen Medien habibi”], B responds: “You already know, who? Not just the German media but all media. This is a known fact.” [“du weißt schon, wer? Nicht nur die deutschen Medien sonder auch alle Medien. Dies ist eine bekannte Tatsache”] (SP-FB[20210511]). By employing world knowledge, anyone who is familiar with this stereotype can infer that it means Jews. B was able to be very clear without committing to this implicit statement.

The idea of a taboo of criticism against Israel is expressed very clearly throughout (“Scary and you can’t say anything about Israel=” [“Gruselig und man darf nix über Israel sagen =”] (B-FB[20210510])—hereafter expressed with the support of a metaphor (“The compliant German NATO press handles Israel with kid gloves. Don’t criticise […]=” [“Die gleichgeschaltete deutsche Nato Presse, fässt Israel mit Sandhanschuhen an. Ja nicht kritisieren […]=”] (FAZ-FB[20210511]).

The distorted portrayals of the conflict in the comments often went as far as giving israel sole guilt for the entire Arab–Israeli conflict. For this, they withhold that the existence of antisemitism and claims to Israeli territory are among the drivers of the conflict within the Palestinian side. These phenomena obviously cannot be part of a reconciliation of interests between the two parties and will therefore continue to stoke the conflict as long as they persist. The range of linguistic realisations reaches from straightforward accusations (“Israel is the culprit in this conflict.” [“Israel ist in diesem Konflikt der Schuldige.”], FAZ-FB[20210511]) to more elaborated comments. With reference to the War of Independence, for example, it is alleged that Israel’s goal would be to drive the Palestinians out of the region and therefore permanent deterrence on the part of the Palestinians would be required: “Defence is important in the Middle East. Otherwise the nakba and flight and expulsion of 1948 would be repeated” [“Verteidigung ist im nahen Osten Wichtig. Ansonsten würde sich die nakba und Flucht und Vertreibung von 1948 wiederholen”] (TAZ-FB[20210512]). Whereas according to this web user, Palestinians acted in a purely defensive manner, the following comment sees all radicalisation as a result of Israeli actions.

“If Israel broke the blockade, withdrew all troops from the West Bank and left the Palestinians alone, then there would be no reason for radicalisation.”

[“Wenn Israel die Blockade auflösen würde, alle Truppen aus der Westbank abziehen würde und die Palästinenser in Ruhe lassen würden, dann gäbe es keinen Grund für Radikalisierung.”] (TAZ-FB[20210512])

The ideas in such comments are often accompanied by statements denying jews the right to self-determination. A very clear statement is: “Palestine under occupation for 73 years” [“Palästina seit 73 Jahren unter Besetzung”] (W-FB[20210511]). Here, the Israeli state territory is declared as *occupation* even at the point it was founded, thereby depriving Israel of its sovereignty over any territory. A more complex variant is the analogy to an absurd fantasy scenario:

“Maybe I should tell the French sometime hey come to Germany Napoleon was here. You can take over the country and anyone resisting gets the death penalty first.”

[“Vielleicht sollte ich mal den Franzosen sagen ey kommt mal nach Deutschland Napoleon war hier. Ihr könnt das Land einnehmen und jeder der sich wehrt bekommt erstmal die Todesstrafe.”] (Z-FB[20210512])

It is used to undermine any claim to a historical connection between Jews and the territory of contemporary Israel. In addition, the image of a tyranny that is depicted here corresponds to a stereotypical attribution of evil.

## Conclusion

We studied user comments on the *Facebook* profiles of leading British, French, and German media outlets on coverage of an escalation phase of the Arab–Israeli conflict. The findings are as varied as they are worrying. Quantitatively, we were able to confirm the observation coming from antisemitism studies that the conflict is a central facilitator for antisemitic communication. Further, it showed that antisemitism was surprisingly high and considerably higher in British comments sections than in French or German ones. In the comments of all three subcorpora, it was noticeable that—in spite of a high percentage of antisemitic contributions making use of linguistic means of implicitness—web users generally do not try to hide the antisemitic meanings behind implicit structures. Rather, antisemitic ideas were expressed openly or with a minimum of subtlety which stands in opposition to our expectations to predominantly find coded forms of antisemitism among the users of the quality media. It seems the users were not under the impression they needed to hide their attitudes. Given the high number of antisemitic comments found, it can be assumed that (with one possible exception) none of the media carried out moderation on their *Facebook* posts. So far, linguistically based research on antisemitism (online) had been mainly carried out for German corpora (cf. e.g., Schwarz-Friesel, 2019a). Findings on linguistic realisations of antisemitism for Germany (concerning implicitness, variations) could be shown in our contrastive analysis in a comparable way for the French and British contexts as well.

The prominently circulated antisemitic concepts presented deny Israel any moral integrity, picture it as an aggressor—whose behaviour would be covered up by pro-Israeli biased reporting—and exclude it from the community of states for a number of alleged reasons.

The qualitatively obtained material discussed here shows clearly how diverse variations of linguistic realisations of antisemitic concepts can occur. This poses a number of challenges when it comes to the identification of such content—first of all for the purpose of moderating it. Apart from the thorough understanding of the conceptual background and characteristics of antisemitism needed, a capability of making adequate inferences concerning the true nature of such content is indispensable—above all when it comes to implicit forms. Those two challenges have to be considered extensively for the implementation of automated moderation tools. Those would have to be trained on data acquired with such a qualitative approach that we presented in order to have the learning basis to recognise such content and to track antisemitic speech on a large scale.

## Data Availability

The datasets generated during and analysed during the current study are available from the corresponding author upon reasonable request.

## Footnotes

1 In other case studies, we focus on all (also non-Israel-related) events that can potentially trigger antisemitic debates. The approach of selecting various events allows us to examine the relationship between the trigger and the content as well as semiotic patterns within the debates online. The annotation system is identical and does not differ from event to event. We have found that—despite differences regarding the language used by commenters—the percentage of antisemitic statements remains fairly constant even in online debates about other antisemitic triggers.

2 The IHRA definition is already used by about 30 states, is furthermore applied at local and regional levels and is recommended for use by the European Union. https://www.holocaustremembrance.com/resources/working-definitions-charters/working-definition-antisemitism. The acceptance is especially crucial since the data gathered in the qualitative analyses will serve as algorithms to learn to detect antisemitism in texts. These algorithms, in turn, will be made publicly available for others (at best even content moderation) in order to scan for antisemitic content and therefore, the decision base of the algorithms has to be approved.

3 In our work, we deal intensively with the grey area that lies between criticism of Israel and Israel-related antisemitism. Using attributions in which Israel is conceptualised as an evil in a temporal framework or across time, we try to elicit context-sensitively the degree of distortion produced by the language used. Insofar as it is a matter of distorting attributions that refer to a limited temporal frame, we annotate this as not antisemitic or overt, in accordance with conservative coding. As soon as Israel is demonised over time or in a quality that excludes the Jewish state and puts it in opposition to the rest of the world, the corresponding comment is annotated as antisemitic.

4 Since stereotypes and other antisemitic topoi are phenomena that exist on the conceptual/mental level and can be reproduced using language, stereotypes are given in small caps on the following pages in accordance with the conventions of cognitive linguistics.

5 https://jerusalemdeclaration.org.

6 The IHRA as well as the JDA are definitions that mainly focus on the content of antisemitic statements. The utterance’s linguistic form plays a secondary role. Exceptions are statements in which “calling for, aiding, or justifying the killing or harming of Jews” (cf. IHRA above) is recognisable. These are speech acts in the form of directives and assertives in which the speaker addresses the non-Jewish in-group and where content and form coincide. Coded forms of antisemitic hate speech (except for labels expressing antisemitic conspiracy theories) are not taken into account.

7 For qualitative research on online antisemitism—among those also corpus studies—see e.g., Iganski and Sweiry (2018), Jikeli et al. (2019), Schwarz-Friesel (2019a, 2019b, 2019c, 2020, 2022), Ozalp et al. (2020), Becker (2021).

8 The users’ national background does not play a major role in our corpus linguistic analysis (and can only be partially assigned due to anonymisation). Rather, for our pattern analysis, it was relevant to investigate what forms of verbal (and visual) antisemitism we could identify on the websites and FB profiles of British mainstream media, which then potentially influence the thinking of other (especially British and UK-based) users. The shift in attention from individuals and groups to the online comments themselves is part of a scholarly approach that looks at the presentation and modification of patterns of prejudice across people and milieus.
